# Saline-tunneling endoscopic intermuscular dissection for the removal of rectal cancer using the hydrodissection method

**DOI:** 10.1055/a-2142-4654

**Published:** 2023-08-21

**Authors:** Felipe Ramos-Zabala, Francisco J. Pérez-Rodríguez, Alejandra Alzina-Pérez, Marian García-Mayor, Luis Moreno-Almazán

**Affiliations:** 1 Servicio de Gastroenterología, Hospital Universitario HM Montepríncipe, HM Hospitales, Boadilla del Monte, Madrid, Spain; 2 Departamento de Ciencias Médicas Clínicas, Facultad de Medicina, Universidad San Pablo-CEU, CEU Universities, Madrid, Spain; 3 Servicio de Anatomía Patológica, Hospital Universitario HM Montepríncipe, HM Hospitales, Boadilla del Monte, Madrid, Spain

A 67-year-old man was referred for consideration of endoscopic versus surgical resection of a rectal polyp after macroscopic suspicion of malignancy on screening colonoscopy. The procedure showed a 25-mm Paris Is + IIc lesion located 3 cm above the dentate line. Within the depression a clearly demarcated area was identified with a distorted pit pattern and irregular vascular pattern ( Fig. 1 ). Staging of the rectal lesion by magnetic resonance imaging and endoscopic ultrasound showed cT1–2N0M0 and uT1N0, respectively. A multidisciplinary decision was taken to proceed with endoscopic intermuscular dissection (EID) 1 for endoscopic local staging and possible definitive management.

Therapeutic endoscopy was performed using saline-tunneling EID with the Erbejet 2 hydrodissection system, an electrosurgical unit (Erbe, Germany) and a colonoscope with transparent hood (Olympus, Japan) ( Video 1 ). To facilitate safe EID, the selective-regulation high-pressure water-jet method was used in the intermuscular space ( Fig. 2 ) 2 . We used only 5 bar water-jet pressure to open across connective tissue in the intermuscular space, which was achieved without damaging the longitudinal muscularis fibers. Saline immersion was used to facilitate visualization of the longitudinal muscularis layer and to obtain optimal countertraction using buoyancy and the distal hood ( Fig. 3 ). We cut the intermuscular space using the T-type hybrid knife probe mode 3 and VIO 3 unit set at PreciseSect mode. The resection was completed within 103 minutes without adverse events. The post-EID defect was not closed ( Fig. 4 ). Pathological examination showed a well-differentiated pT1sm3 adenocarcinoma with low-grade tumor budding, negative for lymphovascular invasion, and with free resection margins ( Fig. 5 ). After multidisciplinary team discussion, the patient decided against completion surgery and intense protocoled surveillance was offered.

**Fig. 1:**
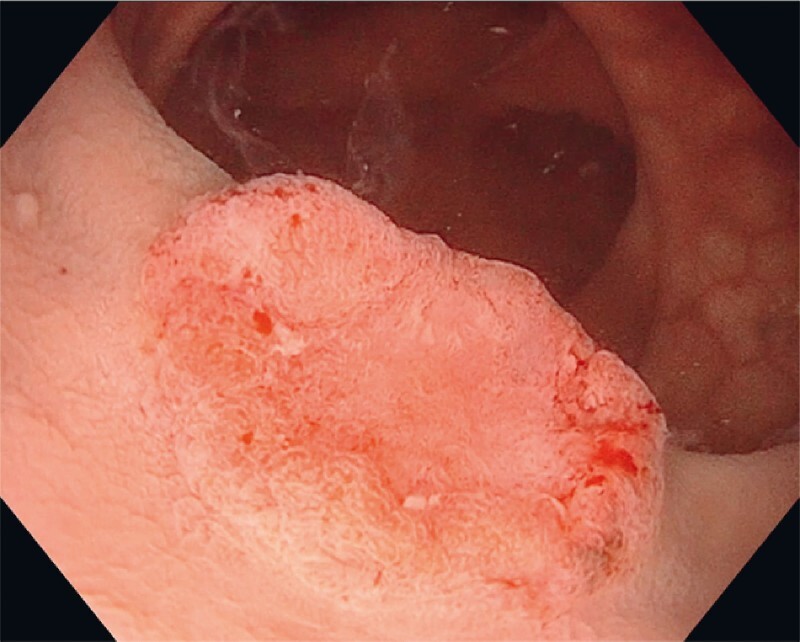
Endoscopic image taken just before endoscopic resection, showing a 25 mm Paris Is + IIc lesion located 3 cm above the dentate line in a 67-year-old man.

**Video 1** Saline-tunneling endoscopic intermuscular dissection for the removal of rectal cancer using the hydrodissection method. Saline immersion and a water-jet of 5 bar was used to open the intermuscular space and facilitate visualization of the longitudinal muscularis layer.


**Fig. 2:**
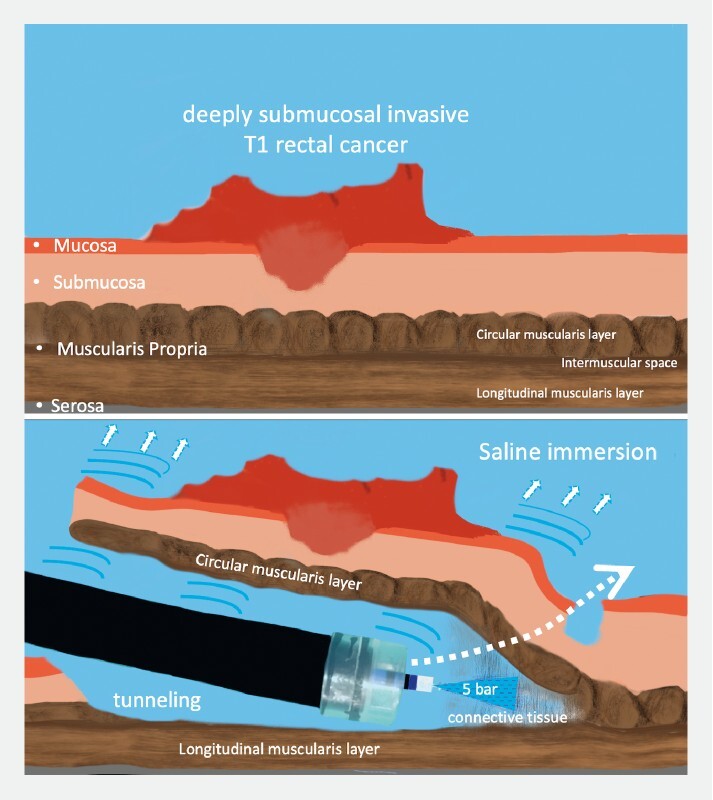
Graphic representation of saline-tunneling endoscopic intermuscular dissection using the hydrodissection method.

**Fig. 3:**
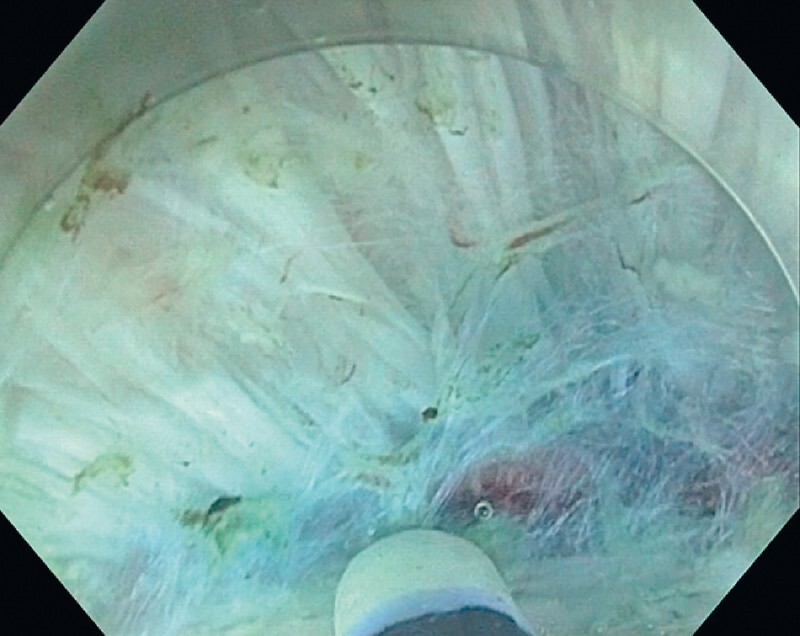
Endoscopic image taken during saline-tunneling endoscopic intermuscular dissection, showing opening of the connective tissue of the intermuscular space facilitated by saline immersion and a water-jet of only 5 bar of pressure.

**Fig. 4:**
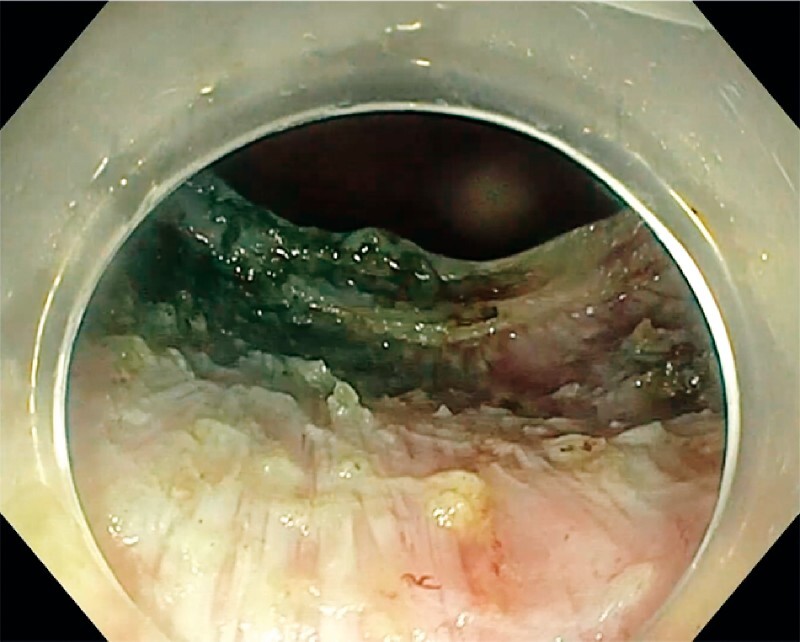
Resection site with visualization of longitudinal muscularis layer after completion of endoscopic intermuscular dissection.

**Fig. 5:**
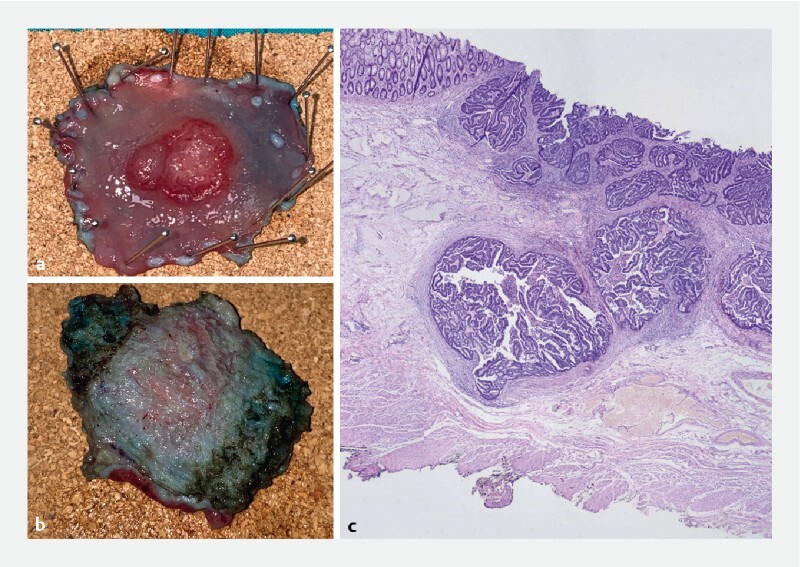
Pathological diagnosis. **a** Resected specimen with rectal polyp with macroscopic suspicion of malignancy. **b** Resected specimen with circular part of the muscularis propria in the deep margin of resection. **c** Histopathologic view of tubular adenoma: well-differentiated pT1Sm3 adenocarcinoma with low-grade tumor-budding, negative for lymphovascular invasion, and with free resection margins. Histopathologic view shows the muscularis fibers of the circular part of the muscularis propria.

Saline-tunneling EID can be a promising endoscopic technique for the resection of deep submucosal invasive T1 rectal cancers. This case report, like previous ones 4 5 , demonstrates that EID procedures greatly facilitate accuracy of early T-staging in well-selected patients.

Endoscopy_UCTN_Code_TTT_1AO_2AG

## Notes

Endoscopy E-Videos

https://eref.thieme.de/e-videos
*E-Videos* is an open access online section of the journal *Endoscopy* , reporting on interesting cases and new techniques in gastroenterological endoscopy. All papers include a high-quality video and are published with a Creative Commons CC-BY license. Endoscopy E-Videos qualify for HINARI discounts and waivers and eligibility is automatically checked during the submission process. We grant 100% waivers to articles whose corresponding authors are based in Group A countries and 50% waivers to those who are based in Group B countries as classified by Research4Life (see: https://www.research4life.org/access/eligibility/ ). This section has its own submission website at https://mc.manuscriptcentral.com/e-videos
